# Stratified Medicine and Reimbursement Issues

**DOI:** 10.3389/fphar.2012.00181

**Published:** 2012-10-15

**Authors:** Hans-Joerg Fugel, Mark Nuijten, Maarten Postma

**Affiliations:** ^1^University of GroningenGroningen, Netherlands; ^2^Ars Accessus MedicaAmsterdam, Netherlands; ^3^FarmacoEpidemiology and FarmacoEconomics, University of GroningenGroningen, Netherlands

**Keywords:** stratified medicine, reimbursement, diagnostics, biomarkers, health technology assessment

## Abstract

Stratified Medicine (SM) has the potential to target patient populations who will most benefit from a therapy while reducing unnecessary health interventions associated with side effects. The link between clinical biomarkers/diagnostics and therapies provides new opportunities for value creation to strengthen the value proposition to pricing and reimbursement (P&R) authorities. However, the introduction of SM challenges current reimbursement schemes in many EU countries and the US as different P&R policies have been adopted for drugs and diagnostics. Also, there is a lack of a consistent process for value assessment of more complex diagnostics in these markets. New, innovative approaches and more flexible P&R systems are needed to reflect the added value of diagnostic tests and to stimulate investments in new technologies. Yet, the framework for access of diagnostic-based therapies still requires further development while setting the right incentives and appropriate align stakeholders interests when realizing long-term patient benefits. This article addresses the reimbursement challenges of SM approaches in several EU countries and the US outlining some options to overcome existing reimbursement barriers for stratified medicine.

## Introduction

Stratified medicine (SM) represents a novel approach to increase pharmaceutical and biopharmaceutical R&D efficiency and to provide improved medical outcomes for the patient and the health care system. Matching therapies to patient populations using clinical biomarker/diagnostics based SM offers the prospect to enhance patient care with safer and more effective drugs, delivered with a greater probability of treatment success. The link between clinical biomarkers and preventive or curative therapies provides new opportunities for value creation, offers the potential to change well-established clinical practices and to strengthen the value proposition to pricing and reimbursement (P&R) authorities.

While the SM approach has drawn great attention in the medical community and the industry, there have been only a few clinical and public health applications in SM to date (Garrison et al., 2007; Blair, 2010; Meckley and Neumann, 2010). Often named examples are: HER2/neu – Herceptin, KRAS/EGFR – Vectibix and Erbitux, predictive for efficacy; UGT1A1/Irinotecan, HLA-5701/Ziagen (HIV), predictive for safety; Oncotype DX and MammaPrint prognostic for adjuvant chemotherapy. Industry is moving slowly to use biomarkers and companion diagnostics in routine clinical practice despite scientific advances and increasing investments in the biomarker-related research and development (Davis et al., 2009; Tufts Center for the Study of Drug Development, 2010). Scientific barriers, concerns about the economic viability of the SM business case and difficulties in securing coverage and adequate reimbursement in various markets are main reasons mentioned.

Although there is a push from health care authorities (FDA^1^, EMA^2^) and payers toward stratification, the application of biomarkers and companion diagnostics to drug development and commercialization is occurring in a complex legal, regulatory, and reimbursement environment. Diagnostics and pharmaceuticals are evaluated by different decision makers within the health authorities, whereas a holistic approach is required in order to assess the full health- and economic value of SM. Third party payers in various healthcare systems have been rather resistant to paying for costly stratification diagnostics unless the diagnostic companies can demonstrate clinical utility and cost-effectiveness without endangering the various health care budgets. At the same time, third party coverage and adequate reimbursement are essential to providing beneficiary access to patient care and to encouraging continued investments in SM interventions.

This paper will discuss the reimbursement challenges of SM approaches in several EU countries and the US. Required changes and options to overcome existing reimbursement barriers for SM will be outlined.

## Concept Stratified Medicine and Reimbursement Challenges

### Concept stratified medicine

**Figure 1:**
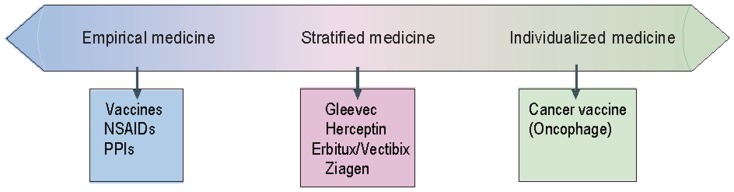
**Stratified medicine diagram (adapted from: Trusheim et al., 2007, The patient therapeutic continuum)**.

Stratified medicine as opposed to empirical medicine is the practice of using biomarkers or diagnostic tests to guide the choice of therapeutic treatments (Trusheim et al., 2007). In the SM case, a predictive diagnostic test stratifies the patient population to responders and non-responders for a certain treatment, whereas by contrast, in empirical medicine all patients would receive the same treatment (Goren, 2007). This approach of proactively testing and selecting populations for specific treatments aims at ensuring increased efficacy and/or reduce toxicity, but at the same time it reduces the eligible patient population. Advances in understanding the mechanisms underlying diseases, as well as drug response, create opportunities to match patients with therapies that are more likely to be effective and safe. At the extreme of patient matching are “individualized” medicines, which vary inherently for each patient such as cancer vaccines that are based on a particular patient’s tumor, representing one end of a continuum (Figure 1).

Empirical medicine is at the other end of this continuum where some agents, such as non-steroidal anti-inflammatory drugs (NSAIDs) work for a large group of patients. In between lies the field of SM, in which a patient can be found to be similar to a cohort that has historically shown a differential therapeutic response to a particular therapy using a clinical biomarker. For example, the anticancer drug trastuzumab (Herceptin) shows superior efficacy in breast cancer patients with HER2/*neu*-positive cancer (expressed in 25–30% of breast cancer patients).

Stratified medicine adds a further step to traditional clinical practice in which a clinical biomarker is evaluated to associate a patient with a specific therapy. The identification of clinical biomarkers or diagnostics linked to the gene expression profile of individual or sub-populations of patients is an essential feature of SM. Trusheim et al. (2007) consider clinical biomarkers to include any diagnostic test or clinical observation that indicates a preferred or contraindicated treatment for a specific patient subpopulation. Such tests can be based on gene expression patterns, individual proteins, proteomic patterns, metabolomics, histology, imaging, physicians’ clinical observations, and even self-reported patient surveys. In other words, they define a clinical biomarker not by its technology or biological basis, but rather by its reliable, predictive correlation to differential patient responses. However, the identification of valid, reproducible associations between genetics, disease progression, and/or treatment response is challenging in the clinical R&D process and implies a good understanding of the molecular mechanisms which causes the disease as well as appropriate studies to identify genetic variants correlated with drug response.

Today, some therapeutic areas include SM approaches and others do not, as at least three specific criteria (Douglas, 2008) will be necessary for the emergence of a clinical relevant patient subclass and consequent SM. These criteria include the presence of: differential biological mechanisms, different treatment options, and a biological marker or diagnostic.

Stratified medicine is practiced in several contexts within the healthcare industry and a shift from “one-size-fits-all” to a tailored approach is already impacting the development of new products. Pharmaceutical and biopharmaceutical industry is using biomarkers as predictors of efficacy and safety to discover new targets and to achieve a differential patient response to therapy helpings to improve the efficiency of compound attrition and R&D productivity over time. Today between 12 and 50% of current clinical pipelines of leading pharmaceutical and biotechnology companies (21 assessed) involves SM, and between 2006 and 2010, the investment of this industry in SM/biomarker research increased by a mean of 75%, with and additional increase of 53% predicted by 2015 (Tufts Center for the Study of Drug Development, 2010). In some cases, industry is developing these markers as companion diagnostic test (e.g., co-developed with a diagnostic company) to identify patient sub-populations most likely to benefit from a particular therapy. For instance, trastuzumab (Herceptin) used a HER2 overexpression test as a predictive marker to increase efficacy in the responder patient population and has achieved blockbusters status. Also, cetuximab (Erbitux) and panitumumab (Vectibix) have benefited from KRAS companion diagnostic test predictive for efficacy in colorectal cancer patients and KRAS testing became mandatory for certain EGFR-kinase-targeted therapies. HLA-B*5701 genetic testing predicts hypersensitivity to abacavir (Ziagen) and found widespread acceptance in the HIV/AIDS treatment to be responsible for the resurgence of this drug in the market. In other cases, SM tests being developed *post hoc* by diagnostic companies as a way of personalizing an existing drug (e.g., metabolic potential of Warfarin to guide dosing) or as stand-alone tests (e.g., Oncotype DX) for diagnostic or prognostic purposes.

### Stratified medicine challenges current reimbursement schemes in EU and the US

Third party payers in public and private health care systems in EU and the US have adopted different P&R policies for drugs and diagnostics. While P&R of pharmaceuticals in many EU countries and the US can be characterized as somewhat “value-based,” the reimbursement of diagnostics is resource or cost-based (Garrison and Austin, 2006). For instance, laboratory-based *in vitro* diagnostic tests have traditionally been treated as low-margin commodity items in many markets with rather low reimbursement rates which are solely based on the method of test (e.g., immunoassay) and not according to the value the tests brings to the patient.

Reimbursement agencies across Europe have compiled lists of devices and procedures which are generally based on the diagnostic-related group (DRG) system (e.g., in Germany G-DRG; France/GHS code). In this system, similar and related medical procedures are grouped together. Each group is then coded and given a monetary value, which is the set amount of money that will be reimbursed for each procedure. In the US, all *in vitro* diagnostic must be assigned to Current Procedural Terminology (CPT) code in order to be reimbursed.

Major payers and other health authorities will make an effort to link new diagnostic to the existing reimbursement level of older tests involving similar effort and cost. This means, that payments must come from an existing budget set for procedure based inpatient DRG’s or linked to out-patients codes set.

To command higher prices for a more complex diagnostic outside the global caps of these procedures will be both challenging and time-consuming in various countries. Currently, there is no clear or consistent process for value assessment of more complex diagnostics established in the EU and the US. Standards and mechanisms such as with AMCP Format in the US and health technology assessment (HTA) with NICE in the UK do not exist in the same way for diagnostics in Western EU countries and the US. Instead, reimbursement of diagnostics in these markets is set on a case-by-case basis where diagnostic tests in most EU countries are reviewed at the local or regional level, and in the US, for example with Medicare, as a combination of national and local jurisdictions.

Only in few examples of companion diagnostic tests, there have been HTA’s in some EU countries (e.g., NICE for HER2, EGFR, and KRAS testing) often followed by cumbersome reimbursement negotiations and resulting in a cost-based funding of the test (Miller et al., 2011). Moreover, in some countries (e.g., UK, Spain) market access for these diagnostic based therapies have been achieved only through subsidizations for the diagnostic tests (lower test prices/or free test) by pharmaceutical manufactures.

There are challenges to determine the clinical and economic value of more complex stand-alone or companion diagnostics. Often there are scientific barriers, such as lack of data that links interventions to health outcomes and costs and that provides comparison to alternative approaches. For a diagnostic test to be useful in clinical practice, it must provide reliable, actionable, and predictive information to a clinician’s treatment recommendation (Deverka et al., 2010). However, in clinical practice, the strength of evidence from simple diagnostic test to rather complex molecular diagnostic test varies widely across types of diagnostics technology. Often, tests are developed to prove clinical validity (sensitivity and specificity of the test) without evaluating clinical utility. Also, case-control, observational, and patient cohort studies are used to determine the clinical value of biomarker based diagnostic when randomized control trials (RCT) are not feasible or too expensive (Scott, 2010). Furthermore, different payers (regional, budget holder) have different evidence requirements (prospective, retrospective) for what is sufficient to determine the clinical utility of a diagnostic test.

There is a significant need to clarify the evidentiary framework of the payers concerning specific characteristics from simple versus complex molecular test for separate development versus co-development. Organizations such as the Center for Medical Technology Policy (CMTP) and the Evaluation of Genomic Applications in Practice and Prevention (EGAPP) in the US as well as the European Personalized Medicine Diagnostics Association are working to explore evidentiary criteria for reimbursement to increase transparency on coverage decisions and to provide industry some guidance when making their decisions to develop innovative diagnostic products (Tunis, 2008). In addition, there are on-going projects from the European Commission such as IT-Future of Medicine (ITFoM) to build a “personalized patient model” and regional initiatives (e.g., DEMOTEK from Basque country) aimed at helping to facilitate the introduction of innovative technologies developed by the local industry.

Directly linked to the quality of clinical evidence there are challenges in determining the economic value of SM interventions. Although many observers have discussed the potential economic value of SM approaches there is currently limited empirical evidence available supporting such claims (Issa, 2007). To date, a few cost-effectiveness analyses exist for SM interventions with inconclusive results as the evidence for effectiveness is frequently preliminary or hypothetical. A recent review by Wong et al. (2010) which examined the economic literature for pharmacogenomics, found 34 economic evaluations where only for two biomarkers there was sufficient evidence supporting both clinical validity and utility, allowing a true cost-effectiveness analysis. The lack of reliable information was also reinforced by a former systematic review of pharmacogenetic and genomic interventions by Vegter et al. (2008) which may explain why currently such cost-effectiveness analyses have little influence on reimbursement decisions in many markets.

To realize the promise of SM approaches there is a need to perform economic evaluations which take into consideration the full impact of using such an intervention on the whole treatment pathway of patients including disease prevention. But in contrast, in many health care systems in Western EU countries and the US there is no longitudinal accounting which would enable payers to capture long-term cost savings from near-term testing. However, anticipated health care costs savings from targeting drug therapy will remain theoretical until a more holistic perspective on healthcare may be followed (Deverka et al., 2010).

Many observers have emphasized the need for more flexible P&R systems which stimulate and reward innovations and reflect the added value of diagnostic tests. Garrison and Austin, see value-based, flexible reimbursement systems for innovative, patent-protected diagnostic being critical to create stronger economic incentives for the development of SM approaches. Seiguer (2007) also concluded that there is hardly any incentive for industry to invest in companion diagnostics unless diagnostics can capture adequate value of diagnostics. Several government commissioned reports have recommended a re-evaluation of reimbursement rates for diagnostics (PCAST, 2008; SACGHS, 2008) by pursuing changes in diagnostic coding and payment systems to better reflect the value of diagnostic tests. However, changing standard coverage principles and/or to establish new coding systems is a rather long-term process in many EU countries and the US.

In the meantime, novel payment approaches, risk sharing, and conditional reimbursement agreements with third party payers are explored to overcome the tension between funding new but expensive technologies and obtaining value for money where traditional reimbursement is not deemed appropriate. These arrangements between a manufacturer and payer/provider can use a variety of mechanisms (e.g., pay-for-performance, value-based purchasing) to address uncertainty about the real performance of technologies in daily practice enabling certain market access. They can help to enhance the value of SM on a case-by-case basis and may provide incentives for the Diagnostic industry to generate high quality clinical and health economic evidence. However, in practice there are many organizational and implementation challenges to overcome to ensure effectiveness of such agreements including the need for a strong collaboration between Pharmaceutical/Biotech and the Diagnostic industry by addressing value sharing issues for companion diagnostic in particular.

Finally, reimbursement authorities are concerned not only with the performance characteristics of new medical diagnostic but also in its feasibility to implement it in a service setting (McCabe et al., 2009). Beside of the availability of appropriate infrastructure, the development, adoption, and the use of medical diagnostics will be influenced by health care provider competence in using these technologies.

## Conclusion and Future Perspective

Stratified medicine can offer the potential to target patient populations who will most benefit from a therapy while reducing unnecessary health interventions associated with side effects and thus, may demonstrate a differential value proposition in order to gain substantial market access. Multiple studies have shown that most drugs prescribed in various diseases are effective in fewer than 60% of treated patients (e.g., oncology only 25–30%) outlining the potential to realize efficiency gains for healthcare systems (Aspinall, 2007).

Medical diagnostics is fundamentally about identifying the subgroups of patients, however, in clinical practice, many of the available tests do not demonstrate clinical utility which makes it difficult to demonstrate the value of diagnostics.

Market Access of SM approaches depends much on the assessment process, in particular HTA and P&R decisions. Today, fragmentation of HTA data requirements and methodology but also of P&R systems for diagnostic testing which are primarily cost/procedure based are not necessarily structured to reward the added value of using tests to improve health outcomes. Novel payment approaches and risk sharing agreement may help to enhance the value of SM interventions on a case-by-case basis provided that clinical and health economics outcomes are transparent.

Generating high quality clinical and health economic evidence will provide the confidence that enables payers more rapidly to adopt tests. At the same time, payer decision making may need to become flexible enough to allow for short-term inefficiencies in order to understand and benefit from long-term value. While the need for market access of diagnostic based therapies is not questions, the framework for access while setting the right incentives and appropriate alignment of stakeholder when realizing long-term patient benefits still needs further development.

Fostering broader coverage of SM approaches within the healthcare systems will require a more centralized, holistic, and consistent process for conducting HTA’s in Europe and the US. Commonly accepted standards and procedures on how to evaluate stand-alone diagnostics and test treatment combinations may provide industry with a clear-cut pathway to market access and reimbursement for population-wide use (Postma et al., 2011). At the same time, a more holistic approach to health care funding is required in order to realize the full clinical and health economic benefits of SM interventions. Because of silo mentality in many health care systems, national authorities may need to develop a central financial system specifically applied for SM interventions.

Recent emerging policy trends and health care financing reform initiatives toward a more value-based healthcare will help to enhance the value of SM approaches in clinical practice. There is a need to take this further and ensure that core SM measures are incorporated into the value-based reimbursement schemes.

## Conflict of Interest Statement

The authors declare that the research was conducted in the absence of any commercial or financial relationships that could be construed as a potential conflict of interest.

## Footnotes

^1^http://www.fda.gov/ScienceResearch/SpecialTopics/CriticalPathInitiative/default.htm

^2^http://www.ema.europa.eu/ema/
